# Using automated active infrared counters to estimate footfall on urban park footpaths: behavioural stability and validity testing

**DOI:** 10.1186/s12966-023-01438-w

**Published:** 2023-04-25

**Authors:** D. J. Ryan, J. S. Benton

**Affiliations:** 1 Centre for Physical Activity and Life Sciences, University of Northampton, Northampton, NN1 5PH UK; 2 Manchester Centre for Health Psychology, Division of Psychology and Mental Health, School of Health Sciences, University of Manchester, Manchester, M13 9PL UK

**Keywords:** Methods, Natural Experiment, Counters, Greenspace, Physical Activity, Walking

## Abstract

**Background:**

Using infrared counters is a promising unobtrusive method of assessing footfall in urban parks. However, infrared counters are susceptible to reliability and validity issues, and there is limited guidance for their use. The aims of this study were to (1) determine how many weeks of automated active infrared count data would provide behaviourally stable estimates of urban park footfall for each meteorological season, and (2) determine the validity of automated active infrared count estimates of footfall in comparison to direct manual observation counts.

**Methods:**

Three automated active infrared counters collected daily footfall counts for 365 days on three footpaths in an urban park within Northampton, England, between May 2021 – May 2022. Intraclass correlation coefficients were used to compare the behavioural stability of abbreviated data collection schedules with total median footfall within each meteorological season (Spring, Summer, Autumn, Winter). Public holidays, events, and extreme outliers were removed. Ten one-hour manual observations were conducted at the site of an infrared counter to determine the validity of the infrared counter.

**Results:**

At least four-weeks (28 days) of infrared counts are required to provide ‘good’ to ‘excellent’ (Intraclass correlation > 0.75, > 0.9, respectively) estimates of median daily footfall per meteorological season in an urban park. Infrared counters had, on average, -4.65 counts per hour (95% LoA -12.4, 3.14; Mean absolute percentage error 13.7%) lower counts compared to manual observation counts during one-hour observation periods (23.2 ± 15.6, 27.9 ± 18.9 counts per hour, respectively). Infrared counts explained 98% of the variance in manual observation counts. The number of groups during an observation period explained 78% of the variance in the difference between infrared and manual counts.

**Conclusions:**

Abbreviated data collection schedules can still obtain estimates of urban park footfall. Automated active infrared counts are strongly associated with manual counts; however, they tend to underestimate footfall, often due to people in groups. Methodological and practical recommendations are provided.

## Background

Restructuring physical environments (such as parks, woodlands and squares) is a promising intervention to increase population-level physical activity. Despite an abundance of cross-sectional evidence between features of the built environment and physical activity levels, there is a dearth of robust intervention-based evaluations [2]. The expectation to conduct robust evaluations of how environmental restructuring increases physical activity participation [20] has grown among Public Health and Government bodies in recent years. This heightened expectation has been particularly evident in England, with the release of policies such as ‘Gear Change: a bold vision for cycling and walking’ [8], ‘Active Travel Fund Monitoring Guidance 2020’ [9], and ‘Improving access to greenspace: a new review for 2020’ [28]. There has also been the establishment of Active Travel England, the executive agency who will act as the inspectorate and funding body for active travel schemes in England [34], as well as cross-Government investment in Green Social Prescription [35], and the launch of [24] Green Infrastructure Framework.

Due to researchers’ lack of control over environmental changes, the optimal study design to evaluate environmental restructuring is to make use of natural experiments. Natural experiments are real-world interventions that are not under the control of researchers and therefore, the exposure to the event or intervention of interest has not been manipulated by the researchers [7]. Researchers can design studies around a natural experiment to assess intervention effectiveness i.e. natural experimental studies.

Within natural experimental studies that have examined changes in physical activity, footfall monitoring is frequently utilised [12]. Footfall monitoring can be conducted using manual counts. However, the use of manual count methods can be resource intensive (i.e. cost of researcher time) and are at risk of sampling error due to the often short-observation window (i.e. four-days, four-hours per day) employed to make causal inferences about the effectiveness of an intervention.

An increasingly popular alternative is to use automated counts from an electronic device. Automated counter systems tend to offer a cheaper and less person-demanding monitoring solution in comparison to manual counts, facilitating longer-term monitoring of interventions. Automated counts are a particularly useful option for local government agencies who often have limited human and financial capacity to conduct evaluations. However, as the market grows for these automated tools, so does the need to assess the reliability and validity for use by researchers and local government agencies. There are several types of automated counters that can be deployed depending on the research question and the environment being studied. Pneumatic tubes have been widely used as a temporary traffic monitoring system, which allows for the distinction between motor vehicles and bicycles. Pneumatic tube systems have demonstrated strong explained variance in comparison to manual observations (*r*^*2*^ = 0.88 – 0.92) but tend to underestimate cycling counts by 6 – 57%, depending on location [16]. Machine Learning Video Camera systems that use publicly accessible traffic cameras have the potential to monitor pedestrians and people cycling in urban environments at scale but are normally limited to dense urban environments, such as town centres, instead of greenspaces [5]. Alternatively, Strava Inc. (San Francisco, USA) released Strava Metro access to local authorities for free during the coronavirus-19 pandemic to facilitate active travel planning. Strava Metro can provide counts along any walked or cycled pathway that is logged by Strava app subscribers, which provides greater flexibility to monitor footfall in any geographical location (greenspaces, urban, remote locations) and have shown moderate to strong correlations with manual observations of cycling in urban environments [15]. In greenspaces, preliminary data has suggested strong correlations between Strava Metro and automated active infrared counters (*r* = 0.75), but there was underestimation path use by 6,639 counts per month [30].

Both automated passive and active infrared counters could be suitable devices for greenspace and rural environment footfall monitoring as they have long battery life, can be easily attached to existing furniture (e.g., gates and fenceposts), and are affordable, although they are unable to distinguish between behaviours (walking and cycling) [18]. Passive infrared counters use a single sensor to detect changes in infrared radiation in their field of view, allowing them to detect humans and animals. Whereas active infrared counters use a gate-system with a transmitter and receiver to create an infrared beam across a path, which identifies the presence of a human or animal when the infrared beam connection between the transmitter and receiver is broken. Thirty-three passive infrared sensors were deployed across Ireland to determine changes in trail-use during the coronavirus-19 pandemic as part of the TrailGazers EU project [27], which aimed to create a framework of technologies to monitor footfall to assist with future planning and tourism management of rural environments and greenspaces. Despite the growing use of infrared counters, they can be susceptible to reliability and validity issues, such as; an inability to count individuals within a group, miscounting due to wildlife or foliage interferences, and vandalism [10]. Furthermore, there is limited guidance for using automated active infrared counters [10, 21].

It is currently unclear how much count data should be collected in order to produce behaviourally stable estimates of urban greenspace footfall especially in different meteorological seasons, due to variations in footfall related to weather conditions. Behavioural stability, a domain of reliability, represents the consistency of a behavioural outcome’s variability over time [13, 31]. Studies have already been conducted to determine the minimum observation days and durations to provide a behaviourally stable estimate footfall with manual observation count tools, such as SOPARC [6] and MOHAWk [3], but no studies, to the author’s knowledge, have been done using infrared counters.

To address these gaps, the aims of this study were to: (1) determine how many weeks of automated active infrared count data would provide behaviourally stable estimates of urban park footfall for each meteorological season, as physical activity levels are known to be highest in spring and summer [33], and (2) determine the validity of automated active infrared count estimates of footfall in comparison to direct manual observation counts. Each aim within the current research was considered under the condition that the automated active infrared counters were fully operational (equipment efficacy).

## Methods

### Research setting

The data collection for the current research took place within Delapré Park, Northampton, England. Northampton is ranked 125^th^ most income deprived, of the 216 Local Authorities in England [25]. Twenty-two of the 133 neighbourhoods within Northampton were categorised as the top 20% most income-deprived in England, while 26 neighbourhoods were within the top 20% least income-deprived [25]. Findings from the 2019 Monitoring Engagement in the Natural Environment Survey suggested that people from Northamptonshire visit greenspaces 90 times per year, similar to the rest of England. They also spend on average 111.8 min per visit within greenspace, which is 25.7 min less than the rest of England [23].

**Fig. 1:**
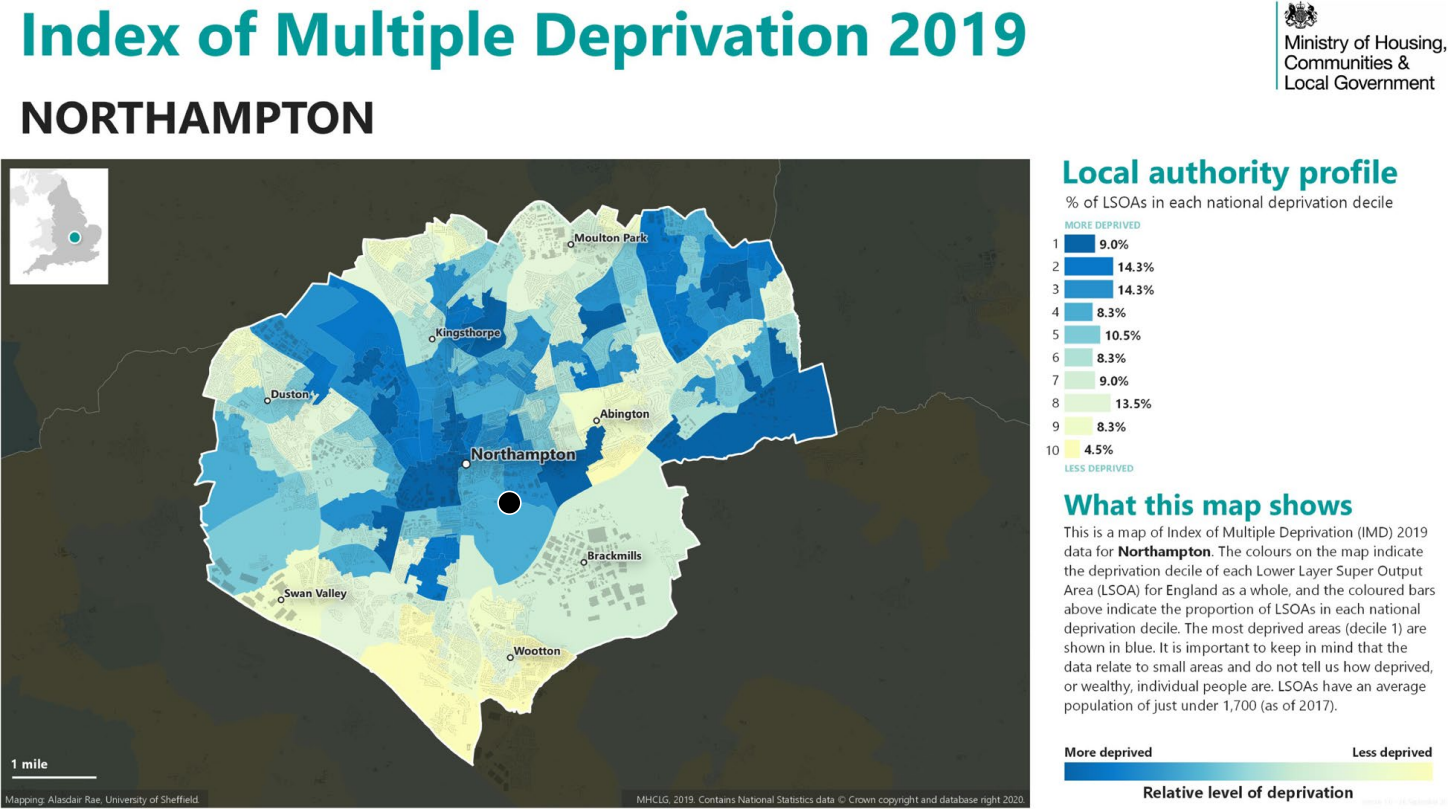
Index of Multiple Deprivation 2019 of Lower Layer Super Output Areas within Northampton, England. Black circle indicates the location of Delapré Park. Map provided freely without required permission by [22]

Delapré Park is located south of Northampton town centre and the River Nene within the urban centre (Fig. 1). This park contains a mixture of land-use, including a Lake, Woods, Heritage Building, and Historic Battlefield. Previous MOHAWk observations within Delapré Park estimated that men (57%) and women (43%) use this park primarily for walking and running (43%) or dog walking (22%) [30]. The majority of park users were observed to be adults (77%) and of white ethnicity (82%) [30]. The park is used by local residents for leisure and active commuting as well as a range of events.

### Automated active infrared counters

**Fig. 2:**
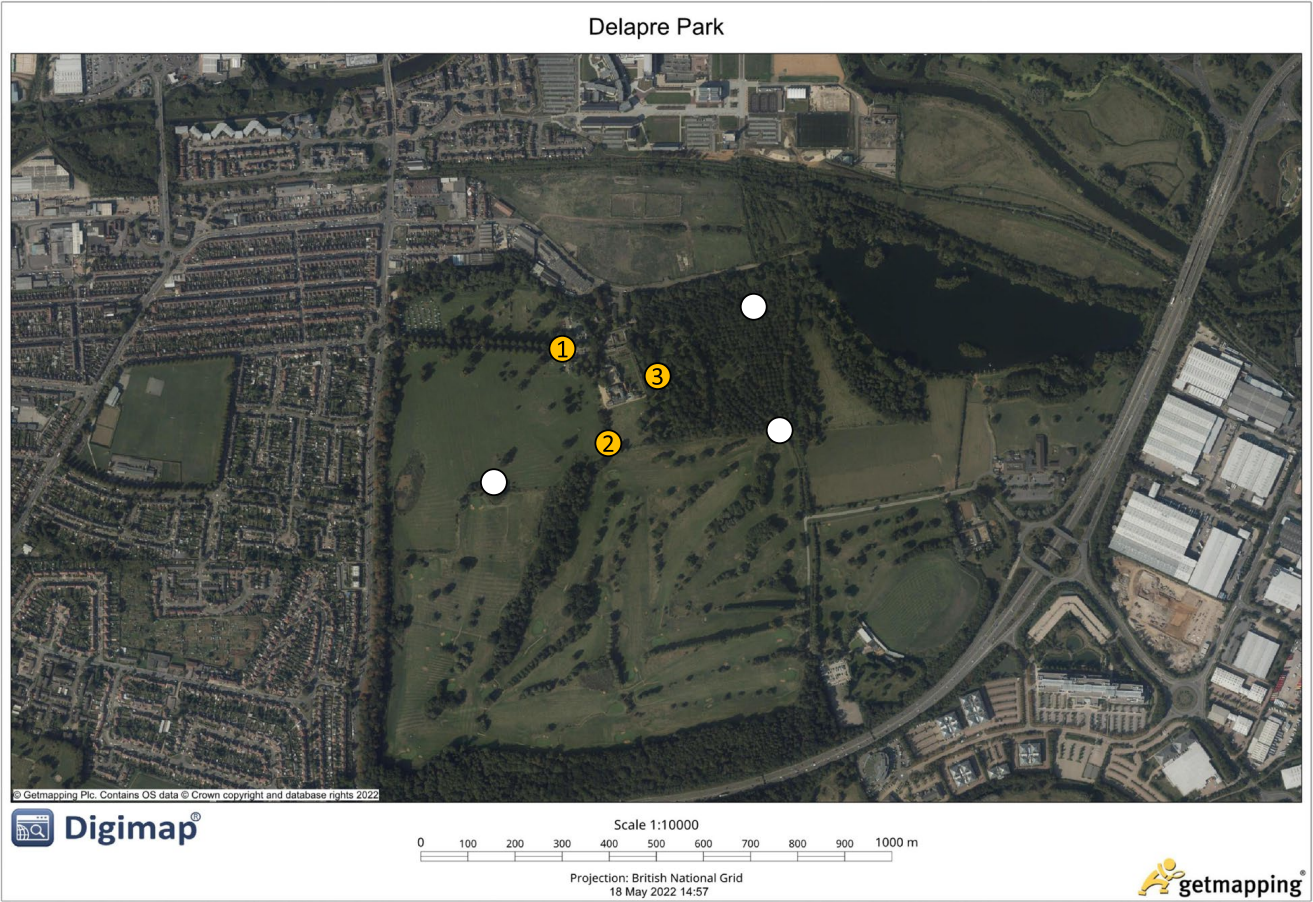
Automated active infrared counter locations within Delapré Park, Northampton, England. Yellow numbered circles indicate counter location and ID number for the counters used within the current study. White circles indicate counter locations as part of the wider project

**Fig. 3:**
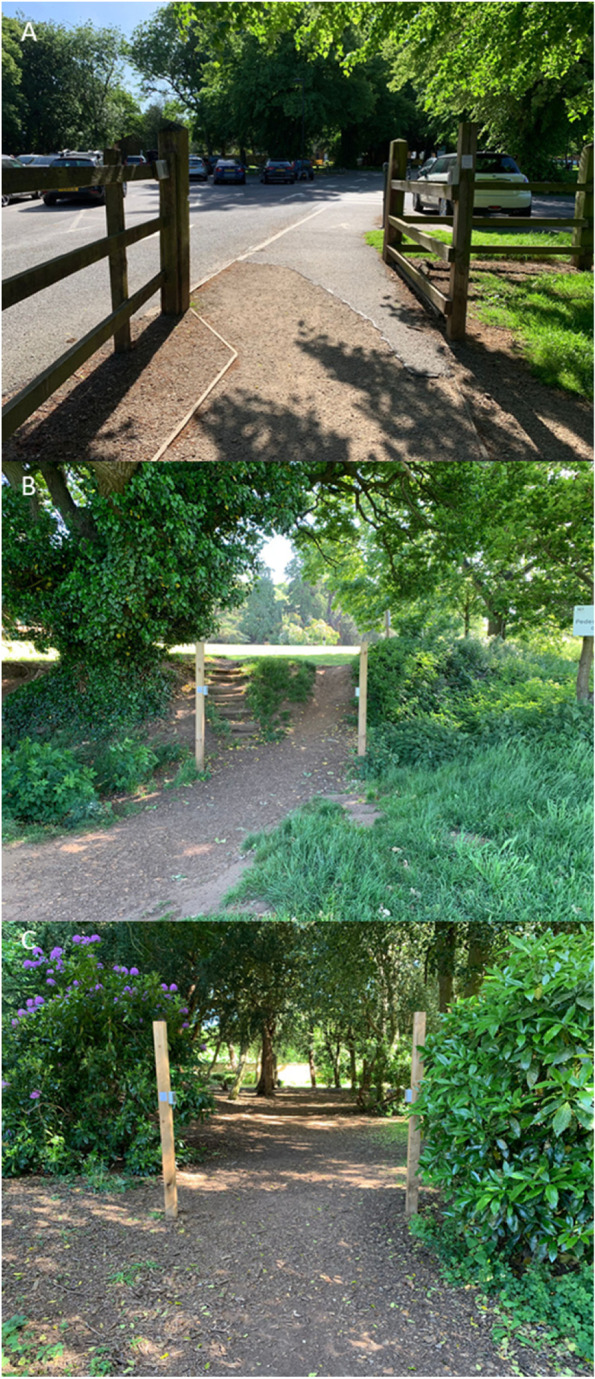
Images of the three counter locations used for the current research. **A** is counter position 1. **B** is counter position 2. **C** is counter position 3

Six automated active infrared counters (DE outdoor bi-directional counter, SensMax Ltd, Riga, Latvia) were installed within Delapré Park as part of a wider project [29]. The counters were placed throughout the park on a 3 km circular walking route (Figs. 2 and 3). For the current study, data from three counters were used as there were no missing data during 365 days of monitoring that was caused by a known fault to the counter, such as mis-alignment of transmitter and receiver units, dead battery, and vandalism. The decision to only use the three counters that had a complete year-long dataset was to increase the generalisability of the findings for researchers or practitioners who may want to use these counters in their own projects. The use of a complete dataset allows the current research to investigate the efficacy of the counters i.e., when the counters are functioning, can they actually estimate seasonal footfall? Within the current study, a day of missing data was usually due to an external issue, such as counter vandalism, a dead battery, or mis-alignment of the transmitter and receiver (this occurred later in the project as the wooden posts, which the counters were attached to, began to warp). If these missing data days were included in the current study, then the findings would reflect counter effectiveness, which is less generalisable for researchers and practitioners as effectiveness is more susceptible to the differing contexts of study locations influencing the findings.

Counters were housed within an ABS plastic with IP68 protection outdoor housing case (SensMax Ltd, Riga, Latvia) and mounted to wooden posts using four wood screws at a height of 1.14 m, which could detect running, walking, cycling, and wheeling behaviours of people over 1.14 m tall, but could not distinguish between these behaviours. The wooden posts for each transmitter and receiver were installed between 2.0 and 2.45 m apart at ‘bottle-neck’ points on footpaths to increase the likelihood of footpath users passing through the counter system (Fig. 3). According to the manufacturer, the counters have a 95% counting accuracy when the transmitter and receiver are placed up to 2.0 m apart, and 1% of accuracy is lost for every additional metre. Data from each counter was downloaded every one-to-two weeks, to minimise data collection disruption due to faults or vandalism, using the SensMax DE Collector remote (SensMax Ltd, Riga, Latvia) and EasyReport 14.1 Pro software (SensMax Ltd, Riga, Lativa).

Counter 1 was located on a paved footpath that was adjacent to the driveway access for the Heritage building. Counter 2 was located at the bottom of a declined grass ramp and steps on the perimeter of the ‘South Lawn’. Counter 3 was located in the ‘Woods’ on a trodden mud path (Figs. 2 and 3). The counters provided counts for each direction of travel on the path from 4^th^ May 2021 to 3^rd^ May 2022. Directional counts were summed to create a total daily footfall for each counter. Total daily footfall was used as the behavioural stability outcome measure for the current research, while directional counts were used for validity testing. Ethical approval was granted by the University of Northampton Faculty Ethics Committee (approval code: 202102).

### Manual observations for counter validation

Counter 1 was chosen to validate the active infrared counters as there was a physical bottleneck at this location, so footpath users had to pass through the counter. Ten one-hour manual observations were conducted by the lead author on Thursday 21^st^ and Friday 22^nd^ July 2022 (five observations per day), starting at 08:00 and finishing at 17:00, with a one-hour break in-between each observation.

The observer recorded the number of people who passed through counter 1, in each direction, during each observation period in order to validate the counts from the automated active infrared counter. The size of groups that passed through the counter was noted, as this has been known to cause underestimations in footfall due to people breaking the infrared beam simultaneously [10]. The sensor cannot capture movement lower than the height of where the infrared beam is placed, so any individuals who were observed to have a height lower than the height of the counter (1.14 m) were noted but were not included in the statistical analysis for this study. This is because the focus of the validation was to determine ‘true counts’ of the sensor, rather than the total footfall of the path. Furthermore, children in pushchairs or being carried by adults were not counted because they would also not be recorded by the sensor. The observation period on 21^st^ July 2022 at 16:00 was abandoned due to a large group of 50 + people passing through the automated active infrared counter, which caused the observer to be uncertain about the total number of people who passed through the counter. Consequently, the 16:00 observation period was repeated on the following week (Thursday 28^th^ July 2022).

### Weather trends

To outline trends in weather conditions across meteorological seasons, daily mean temperature (degrees centigrade), daily mean wind speed (miles per hour), and daily total rainfall (inches) were monitored from a local weather station [37]. These real-time measures of weather were recorded as they have previously been associated with various measurements of physical activity [4].

### Statistical analysis

#### Behavioural stability of abbreviated observational periods

For each counter, days determined to have extreme count outliers (i.e. a value more than three times the inter-quartile range (Q3–Q1) from the upper (Q3) or lower (Q1) quartile) were omitted from the analysis. To determine an explanation for each outlier, these outlier days were cross-referenced with dates of public holidays in England and site-specific events hosted at the park (e.g. a one-off running event), because the Heritage Building located within the park had historically experienced increases in footfall on these days. Any counts that occurred on public holidays were omitted from analysis for all three counters, whereas counts on event days were only omitted from the analysis for counters that reported an extreme count outlier on that day. These decisions were made to minimise the influence of inflated counts and improve the generalisability of the findings as other parks that may not host events or the country of the park may have different public holidays. All remaining extreme outliers were greater than the mean counts per day and were thus thought to be due to either foliage obscuring the counter, or purposeful tampering with a counter (e.g. waving a hand to break the beam frequently). This resulted in a total of 328 days (Counter 1), 339 days (Counter 2) and 341 days (Counter 3) of monitoring that were retained for analyses. The handling of outliers was made as the authors expected that these counters may be used in natural experimental studies or longitudinal monitoring of park footfall and within these projects, the inclusion of counts from public holidays or events could present an overinflation of park footfall and therefore, may be omitted or adjusted for. Furthermore, the decision to remove extreme outliers that were not due to public holidays or events was informed by author knowledge of the area through the hours of observations accrued by visiting the park throughout the study. This development of researcher knowledge of the study area aligns with recommended planning processes for natural experimental studies and the use of manual observation tools [2, 3].

Two-way mixed, single measure, consistency intraclass correlation coefficients (ICCs) were used to calculate the mean behavioural stability of total median counts of each counter for different abbreviated data collection schedules within each meteorological season (Spring, Summer, Autumn, Winter). Specifically, mean ICCs were calculated for all possible combinations of 1, 2, 3 and 4-weeks per season for each counter: there were 13 unique combinations for 1-week (as each season had a total of 13 weeks); 78 unique combinations for 2-weeks; 286 unique combinations for 3-weeks; and 715 unique combinations for 4-weeks. Weeks that included days of missing data were omitted from analysis as this would have caused incomplete data being used to calculate mean ICCs (e.g. only 3-weeks of data in a 4-week combination). The mean ICCs were then compared to the entire season. ICCs can be interpreted as  < 0.5 = poor; 0.5 – 0.75 = moderate; 0.76 – 0.9 = good; and  > 0.9 = excellent [14].

No adjustment for weather was made in the behavioural stability analyses because the weather is closely associated with meteorological seasons, and therefore segregating analyses into meteorological seasons would sufficiently account for weather variability. Analyses were performed using SPSS Statistics version 28.0 (IBM, New York, USA).

#### Validity of automated active infrared counters

Bland–Altman plots [1] were used to determine convergent validity of the automated active infrared counter in comparison to manual observation. Directional counts were used as separate data points and thus 10 observation periods × two directions of counts = 20 data points for analysis. A one-sample t-test was used to determine systematic bias in the difference between the two count methods. Limits of Agreement were calculated by multiplying the standard deviation of the difference by 1.96. A linear regression was used to determine proportional bias in the difference between the two count methods. Finally, a linear regression was conducted to determine the concurrent validity of hourly automated active infrared counts for hourly manual observation counts.

A forced entry single linear regression was used to determine whether the total number of groups that passed through a counter predicted the amount of difference between automated active infrared counts and manual observation counts.

## Results

### Weather trends

The average weather trends for each season are provided in Table 1, which have similar ranges to 1991 – 2020 climate periods for the area [19].

**Table 1:** Weather descriptive statistics for each meteorological season

	**Season**			
**Weather**	**Spring**	**Summer**	**Autumn**	**Winter**
Daily mean temperature (˚C)	9.74 ± 2.88 (2.55 – 17.1)	17.5 ± 2.39 (12.7 – 24.6)	13.1 ± 4.88 (0.44 – 22.3)	6.64 ± 2.97 (1.11 – 14.0)
Daily total rainfall (in)	0.04 ± 0.16 (0.00 – 0.96)	0.07 ± 0.21 (0.00 – 1.53)	0.05 ± 0.15 (0.00 – 0.90)	0.08 ± 0.11 (0.00 – 0.45)
Daily mean wind speed (mph)	2.56 ± 1.35 (0.50 – 7.50)	2.01 ± 1.11 (0.30 – 5.10)	2.15 ± 1.28 (0.00 – 5.60)	3.32 ± 1.96 (0.20 – 8.40)

Displayed as mean ± standard deviation (minimum – maximum)

### Behavioural stability of abbreviated observational periods

Table 2 displays the mean ICCs and 95% Confidence Intervals (95% CI) for 1, 2, 3 and 4-week combinations for each counter, in comparison to meteorological season median daily counts. On average, for all three counters, collecting data on 4-weeks for each season can produce good or excellent consistency for median daily counts approaching that obtained by collecting data for the entire meteorological season. Autumn and Spring tended to require fewer weeks to obtain at least good consistency in comparison to the entire season (e.g. Counter 2 obtained good behavioural stability with just one-week) and had narrower confidence intervals, compared with the one-week for Summer and Winter.

**Table 2:** Behavioural stability estimates using the mean ICC for 1, 2, 3 and 4-weeks compared with the entire meteorological season for median daily counts

Counter Location	Season (number of days included for analysis)	Median count per day (IQR)	Number of weeks			
			**1**	**2**	**3**	**4**
			**Mean ICC (95% CI), Number of combinations used to calculate the mean**	**Mean ICC (95% CI), Number of combinations used to calculate the mean**	**Mean ICC (95% CI), Number of combinations used to calculate the mean**	**Mean ICC (95% CI), Number of combinations used to calculate the mean**
Counter 1	Spring (81 days)	237 (118)	0.59 (-0.16—0.91), 10	0.73 (0.07—0.95), 45	0.71 (0.04 – 0.94), 120	0.80* (0.24 – 0.96), 210
	Summer (87 days)	298 (114)	0.46 (-0.35—0.88), 11	0.63 (-0.09—0.92), 55	0.64 (-0.07 – 0.93), 165	0.76* (0.15 – 0.95), 330
	Autumn (87 days)	231 (95)	0.79* (0.22—0.96), 9	0.88* (0.50—0.98), 36	0.84* (0.36 – 0.97), 84	0.90** (0.56 – 0.98), 126
	Winter (73 days)	204 (100)	0.42 (-0.28—0.84), 8	0.63 (-0.05—0.92), 28	0.70 (0.15 – 0.93), 56	0.82* (0.37 – 0.96), 70
Counter 2	Spring (81 days)	129 (65)	0.77* (0.21—0.95), 10	0.88* (0.50—0.98), 45	0.91** (0.57 – 0.98), 120	0.95** (0.74 – 0.99), 210
	Summer (86 days)	139 (45)	0.55 (-0.20—0.90), 11	0.71 (0.06—0.94), 55	0.80* (0.29 – 0.96), 165	0.88* (0.48 – 0.98), 330
	Autumn (90 days)	122 (67)	0.80* (0.32—0.96), 12	0.89* (0.55—0.98), 66	0.91* (0.61 – 0.98), 220	0.95** (0.74 – 0.99), 495
	Winter (82 days)	101 (47)	0.66 (0.05—0.92), 10	0.81* (0.33—0.96), 45	0.83* (0.36 – 0.97), 120	0.90** (0.57 – 0.98), 210
Counter 3	Spring (80 days)	93 (54)	0.78* (0.19—0.96), 9	0.89* (0.51—0.98), 36	0.89* (0.53 – 0.98), 84	0.94** (0.69 – 0.99), 126
	Summer (88 days)	96 (44)	0.51 (-0.21—0.86), 12	0.64 (-0.04—0.92), 66	0.73 (0.14 – 0.94), 220	0.81* (0.32 – 0.96), 495
	Autumn (91 days)	81 (46)	0.71 (0.12—0.94), 13	0.85* (0.42—0.97), 78	0.85* (0.44 – 0.97), 286	0.91** (0.61 – 0.98), 715
	Winter (82 days)	57 (40)	0.63 (-0.06—0.92), 10	0.76* (0.17—0.95), 45	0.73 (0.11 – 0.95), 120	0.81* (0.29 – 0.96), 210

Good’ (ICC > 0.75) or ‘excellent’ (ICC > 0.9) behavioural stability scores are indicated with bold font and a * or ** respectively. 91 days was the maximum number of days available for each season. CI – confidence interval. There was a maximum of 13 unique combinations for 1-week (as each season had a total of 13 weeks); 78 unique combinations for 2-weeks; 286 unique combinations for 3-weeks; and 715 unique combinations for 4-weeks

### Validity of automated active infrared counters

**Fig. 4:**
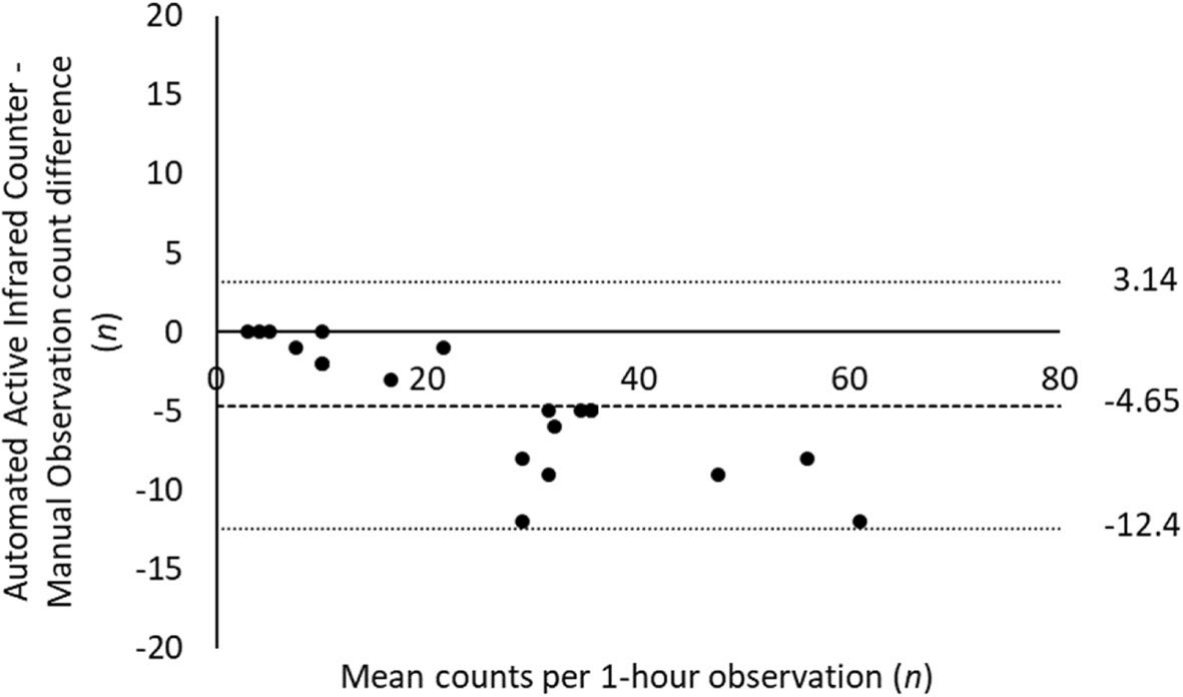
Bland–Altman plot displaying the difference between automated active infrared and manual observation counts across one-hour observation periods. Scatter points represent a directional count for one-hour observation periods (two directional counts per observation period). Dashed line represents the mean difference between automated active infrared and manual observation counts across one-hour observation periods, while dotted lines represent the 95% Limits of Agreement. *P* < 0.001

**Fig. 5:**
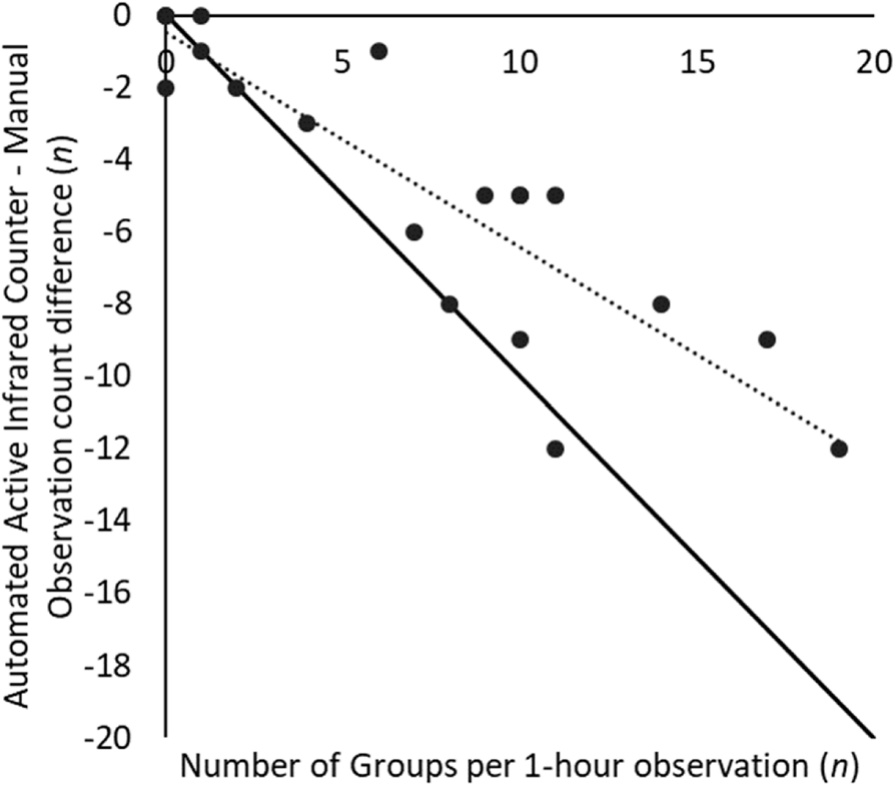
Relationship between the number of groups per one-hour observation period and the difference between automated active infrared and manual observation counts. Scatter points represent a directional count for one-hour observation periods (two directional counts per observation period). Dashed line represents the linear trendline (*r*^2^ = 0.78, *p* < 0.001). Solid line represents the line of unity

**Fig. 6:**
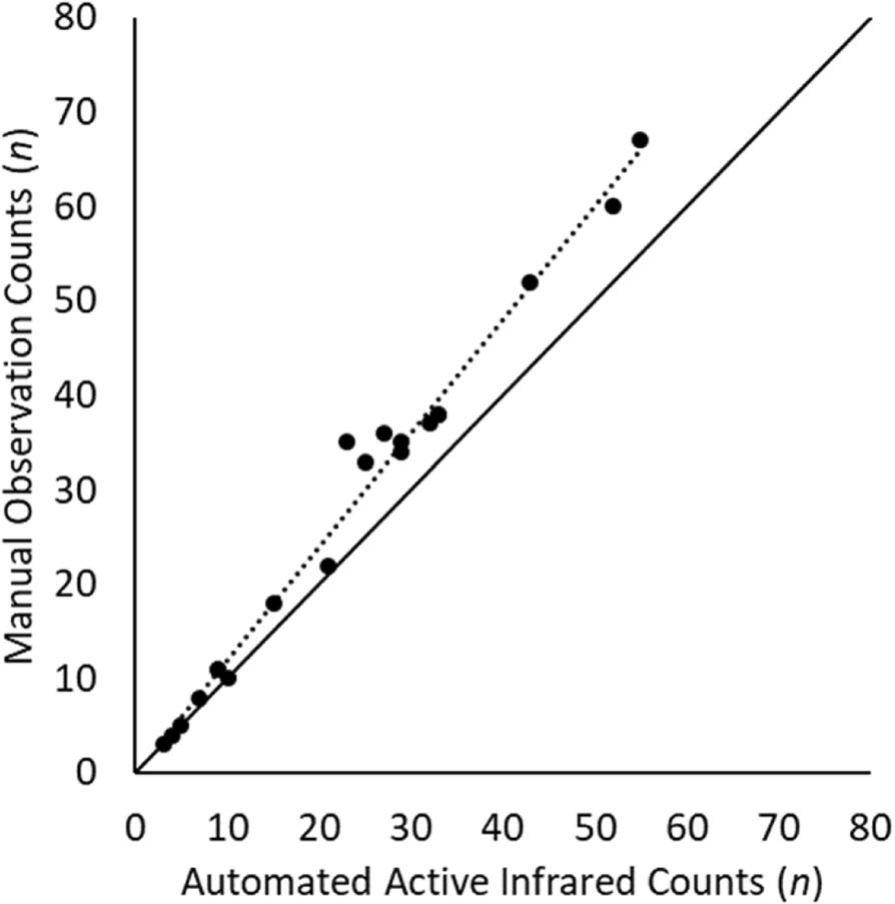
Relationship between automated active infrared counts and manual observation counts. Scatter points represent a directional count for one-hour observation periods (two directional counts per observation period). Dashed line represents the linear trendline (*r*^2^ = 0.98, *p* < 0.001). Solid line represents the line of unity

Manual observation counts during the ten one-hour observation periods ranged from 3 to 67 counts, with the number of groups per observation period ranging from 0 to 19 groups. Automated active infrared counters were, on average, -4.65 counts per hour (95% Limits of Agreement -12.4, 3.14 counts, *p* < 0.001, Mean absolute percentage error 13.7%) lower than manual observation counts per one-hour observation period (23.2 ± 15.6, 27.9 ± 18.9 counts per hour, respectively, 16.7 percentage difference), demonstrating systematic bias. There was a negative association between mean counts per one-hour observation and the difference between automated active infrared and manual observation counts (β -0.19, 95% CI -0.26, -0.13 counts, *p* < 0.001, intercept 0.31, *r*^*2*^ = 0.71), suggesting proportional bias (Fig. 4). The number of groups per one-hour observation period explained 78% (*r*^*2*^ = 0.78) of the variance in the difference between automated active infrared and manual observation counts (β -0.60, 95% CI -0.75, -0.44 counts, *p* < 0.001, intercept -0.48; Fig. 5). Automated active infrared counts explained 98% (*r*^*2*^ = 0.98) of the variance in manual observation counts per one-hour observation period (β 1.21, 95% CI 1.13, 1.28 counts, *p* < 0.001, intercept -0.096; Fig. 6).

## Discussion

### Summary of key findings

The behavioural stability study found that four-weeks (28 days) of automated active infrared count data are required to estimate the median daily count for a meteorological season. The validation study indicated that automated active infrared counts are strongly associated (*r*^*2*^ = 0.98) with manual counts, albeit underestimating on average by -4.65 counts per one-hour observation period (Mean absolute percentage error 13.7%). The number of groups passing through a counter explained 78% of the variance in the difference between automated active infrared and manual counts, suggesting that automated active infrared counters struggle to identify group sizes.

### Comparisons with other count methods

Previous monitoring of outdoor physical activity in parks, which used automated infrared counters to monitor footfall, have used a variety of weeks for data collection. Natural experimental studies, where a pre-post comparison was used, collected count data over periods of 19-weeks (five-months) [11] to eight-days [36] per time point. Meanwhile, a validity study for automated active infrared counters (TrailMaster TM1550, Lenexa, USA) to estimate footfall in Yosemite National Park, USA, used a 122-day observation period [26]. Trend monitoring studies have also employed varying lengths of observation periods ranging from two-months [17] to two-years [27]. Therefore, there seems to be little consensus on how many monitoring days per observation period is enough to provide estimates of footfall. The current study has begun the process of establishing a recommended minimum observation period for meteorological seasonal footfall estimates when using a SensMax DE active infrared counter (SensMax Ltd, Riga, Latvia). This is important because while it may be feasible for municipalities, national parks, or large-funded research projects to conduct continuous year-round monitoring of footfall, the costs of both labour and time resources associated with infrared counters may make continuous monitoring untenable for local government agencies. More research is required using counters from different manufacturers and different study locations (geographical, topography, climate, and socio-cultural contexts) before a generalisable consensus on infrared counter behavioural stability can be attained. Notably, in the validation data collection the SensMax DE only underestimated counts, which is due to the binary classification (count or no count) provided by this counter. Theoretically, the SensMax should only underestimate counts but, in the field, overestimation may occur due to foliage, large animals, or people intentionally breaking the infrared beam. Therefore, researchers should consider mitigation approaches to reduce these risks to overestimation of counts.

The current study found that SensMax DE bi-directional automated active infrared counters (SensMax Ltd, Riga, Latvia) underestimated total counts (-4.65 counts per one-hour observation period, Mean absolute percentage error 13.7%). This systematic bias represented a -16.7 percentage difference between automated active infrared counts and manual counts, regardless of the direction of travel, which is higher than integrated passive infrared inductive loop counters (-10.3 percentage difference in pedestrian counts; Eco-Multi Sensor, EcoCounter, Lannion, France) that have also undergone a 10-h validation comparison against manual counts [16] and have been used recently in a park natural experiment evaluation [11]. However, the SensMax DE automated active infrared counter does display a similar explained variance of manual counts (*r*^*2*^ = 0.98) in comparison to the Eco-Multi Sensor (*r*^*2*^ = 0.91 – 0.99) [16]. In comparison to the Trail-Master 1500 active infrared counter (Trail-Master, Lenexa, USA), the SensMax counter from the current study displays a similar explained variance of manual counts (SensMax: *r*^*2*^ = 0.98, Trail-Master: *r*^*2*^ = 0.99), as well as a similar regression coefficient gradient (SensMax: β 1.21, Trail-Master: β 1.15) [17]. Comparisons can also be drawn from the Trail-Master 1150 model (Trail-Master, Lenexa, USA) large-scale calibration study in Yosemite National Park, USA, which estimated an explained variance of manual counts between 0.96 – 0.99 and a regression coefficient gradient of 1.57 – 1.83 [26]. Therefore, the SensMax DE bi-directional automated active infrared counter (SensMax Ltd, Riga, Lativa) performs similarly to other infrared counter types that have been previously used to estimate footfall in parks. Thus, the findings of the current study suggest the SensMax DE can provide valid estimates of footfall and is recommended for use in park footfall monitoring research.

### Practical considerations when using automated counters

Based on this study, there are a number of practical recommendations for researchers using automated counters. Automated counters often have substantial battery life; the SensMax DE counter uses two AA batteries and has a battery life of over one-year, including a memory to record up to 150 days of data, which means they can be deployed over long periods without maintenance by researchers. However, it is recommended that weekly site visits are conducted to download the data and check the counters for vandalism or disturbances (e.g. the counter peeling off the double-sided adhesive attachment in the outdoor housing case). In the current study, the sturdy outdoor housing case and the use of four woodscrews to fix the outdoor housing case to fenceposts made it difficult for vandalism and unintended damage to the counters. Across one-year of monitoring, there were only six cases of counter vandalism or damage. However, it is highly likely that vandalism will occur to counters, so researchers should have a contingency budget to replace and repair counters. Practical applications to reduce the risk of vandalism include: (1) embedding the outdoor housing case within the structure that it is attached to so, a saw cannot reach the woodscrews, the presence of the counters are less noticeable, and only the front of the case could be hit with an object, (2) ensure posters are erected around the monitoring site so visitors are informed that counting is occurring as well as what data is being collected, (3) promotion of the research to establish community awareness of the project, which can encourage visitors to act as a self-policing community who will deter vandals and report any suspicious activity to the researchers or police.

Furthermore, caution needs to be used if the counters are attached to wooden fence posts as the posts can warp during changing weather conditions, which causes the counters’ infrared beams to misalign. If misalignment of counters does occur due to the fixing site changing shape, then it is possible to realign the counters by changing the position of the counter within the outdoor housing case or loosening the woodscrews to change the angle of the outdoor housing case.

### Strengths and limitations

A strength of this study was the use of year-long automated active infrared counter data from three separate counters, which provided up to 1,095 days of count records. This provided a large sample, therefore offering reassurance that the findings of this study are representative of the entire year and not due to sampling error. This study also demonstrates the effect of group presence on automated count bias, which had been previously assumed but not formally assessed [10]. We have provided new methodological and practical recommendations for researchers using automated active infrared counters in the growing field of natural experimental studies for urban environment interventions on outdoor physical activity.

The main limitation of this study is that data collection began in May 2021, which was during the coronavirus-19 pandemic. At this time point, England had entered Step 2 of coronavirus-19 lockdown easing. Non-essential retail and outdoor venues had reopened, but no indoor mixing between different households was allowed. These restrictions varied over the course of the study period. For instance, on 19^th^ July 2021, most legal limits on social contact were removed and the final closed sectors were reopened [32]. Yet on 10^th^ December 2021, England entered ‘Plan B’ restrictions by making face masks and contact tracing compulsory in most indoor settings and encouraging people to work from home [35]. Although there is nothing the researchers could do to overcome this limitation, it is likely that changes in coronavirus-19 restrictions and public opinions of the virus had some influence on the use of outdoor spaces [27] and the recorded counts in the current study. However, even with these potential fluctuations in counts caused by responses to coronavirus-19, the current study still demonstrated that only four-weeks of automated active infrared counts were needed to provide an estimate of median daily counts per meteorological seasonal. If footfall is presumed to be more consistent across the year without the effects of coronavirus-19 restrictions, then this may lead to fewer weeks of count data needed to obtain seasonal estimates. Furthermore, the authors made justified decisions regarding the handling of outliers however, the generalisability of the resultant findings may be limited depending on how future studies design their data collection and analysis protocol, such as the inclusion of public holidays, events, extreme outliers in their data sets. Population monitoring of physical activity in public park settings is subject to physical and socio-cultural contexts that are unique to the study location. Therefore, replication studies are required in differing contexts to build a consensus of how many weeks of infrared counter monitoring are required, in what contexts, to provide behaviourally stable estimates of footfall for each meteorological season.

## Conclusion

This study provided novel insights into the application of automated active infrared counters for footfall monitoring in parks. Findings suggested that at least four-weeks of automated active infrared counter data was required to provide estimates of median daily counts per meteorological season in an English urban park. Even though automated active infrared counts underestimated manual counts, they were still strongly associated. The main cause of automated active infrared count error was due to the presence of groups walking through the counters. Further research is needed to provide behavioural stability estimates and validation of automated active infrared counters in different climates, localities, and socio-cultural contexts to build a robust evidence base that informs the appropriate use of infrared counters in different contexts.

## Data Availability

The dataset supporting the conclusions of this article is available in the University repository, Pure, https://pure.northampton.ac.uk/en/datasets/using-automated-active-infrared-counters-to-estimate-footfall-on-; https://doi.org/10.24339/3ef61812-75c7-4431-8423-7358d0c296f2.

## Abbreviations

CI: Confidence intervals
ICC: Intraclass correlation coefficient
IQR: Interquartile Range
MOHAWk: Method for Observing pHysical Activity and Wellbeing
Q: Quartile
SOPARC: System for Observing Play and Recreation in Communities
